# Increased Visceral Adipose Tissue as a Potential Risk Factor in Patients with Embolic Stroke of Undetermined Source (ESUS)

**DOI:** 10.1371/journal.pone.0120598

**Published:** 2015-03-10

**Authors:** Antti T. Muuronen, Mikko Taina, Marja Hedman, Jarkko Marttila, Johanna Kuusisto, Juha Onatsu, Ritva Vanninen, Pekka Jäkälä, Petri Sipola, Pirjo Mustonen

**Affiliations:** 1 Kuopio University Hospital, Diagnostic Imaging Centre, Department of Clinical Radiology, Kuopio, Finland; 2 Kuopio University Hospital, Heart Center, Kuopio, Finland; 3 Kuopio University Hospital, Department of Medicine, Kuopio, Finland; 4 Kuopio University Hospital, Neuro Center, Kuopio, Finland; 5 University of Eastern Finland, Institute of Clinical Medicine, Unit of Radiology, Kuopio, Finland; 6 University of Eastern Finland, Institute of Clinical Medicine, Unit of Neurology, Kuopio, Finland; 7 Department of Cardiology, Keski-Suomi Central Hospital, Jyväskylä, Finland; Innsbruck Medical University, AUSTRIA

## Abstract

**Purpose:**

The etiology of an ischemic stroke remains undetermined in 20–35% of cases and many patients do not have any of the conventional risk factors. Increased visceral adipose tissue (VAT) is a suggested new risk factor for both carotid artery atherosclerosis (CAA) and atrial fibrillation (AF), but its role in the remaining stroke population is unknown. We assessed the amount of VAT in patients with embolic stroke of undetermined source (ESUS) after excluding major-risk cardioembolic sources, occlusive atherosclerosis, and lacunar stroke.

**Methods:**

Altogether 58 patients (mean age 57.7±10.2 years, 44 men) with ischemic stroke of unknown etiology but without CAA, known AF or small vessel disease underwent computed tomography angiography and assessment of VAT. For comparison VAT values from three different reference populations were used. Conventional risk factors (smoking, hypertension, diabetes, increased total and LDL-cholesterol, decreased HDL-cholesterol) were also registered.

**Results:**

Mean VAT area was significantly higher in stroke patients (205±103 cm^2^ for men and 168±99 cm^2^ for women) compared to all reference populations (P<0.01). 50% of male and 57% of female patients had an increased VAT area. In male patients, VAT was significantly higher despite similar body mass index (BMI). Increased VAT was more common than any of the conventional risk factors.

**Conclusion:**

Increased VAT was found in over half of our patients with ESUS suggesting it may have a role in the pathogenesis of thromboembolism in this selected group of patients.

## Introduction

Stroke is the leading cause of long-term disability, the second leading cause of death and a major consumer of healthcare resources worldwide [1–4]. Ischemic stroke accounts for 80% of all strokes in the Western countries and is mostly caused by atheroma of the cervical arteries, embolization from intracardiac sources, or by microvascular disease [5]. In 20–35% of the cases the etiology of stroke remains undetermined (cryptogenic) despite profound investigations [6, 7]. Patients with embolic stroke of undetermined source (ESUS) are a subset of patients with cryptogenic stroke who have embolic strokes and sufficient diagnostic assessment to exclude major-risk cardioembolic sources, occlusive atherosclerosis, and lacunar stroke [8].

Obesity is a well-known risk factor for all cardiovascular diseases and stroke, and it increases the risk of all-cause mortality [9]. A traditional way to define obesity is to measure body mass index (BMI) [10, 11]. Recently, more attention has been paid to the distribution of the adipose tissue in the body. The increased visceral fat type in particular has been stated to increase the risk of stroke and other cardiovascular diseases [12–16]. Adults with visceral adiposity tend to manifest insulin resistance, hypertension and dyslipidemia more often when compared to those who are equally obese with lower levels of visceral fat [17, 18]. The aforementioned metabolic changes have been postulated to cause a hypercoagulable state both in the arterial and venous system and, consequently, play a role in the pathogenesis of stroke [19]. Visceral fat can be measured accurately with computed tomography (CT) [20, 21]. To the best of our knowledge, no previous study has investigated the prevalence of VAT in a population with ESUS, i.e. in which atherosclerosis, small vessel disease or known AF are excluded as stroke etiologies.

## Materials and Methods

The study was approved by the Kuopio University Hospital Research Ethics Board and all clinical investigations have been conducted according to the principles expressed in the Declaration of Helsinki. Prior to participation in the study, written informed consent was obtained from the patient or the patient's legally authorized representative.

### Study patients and reference populations

#### Study patients

**Fig 1:**
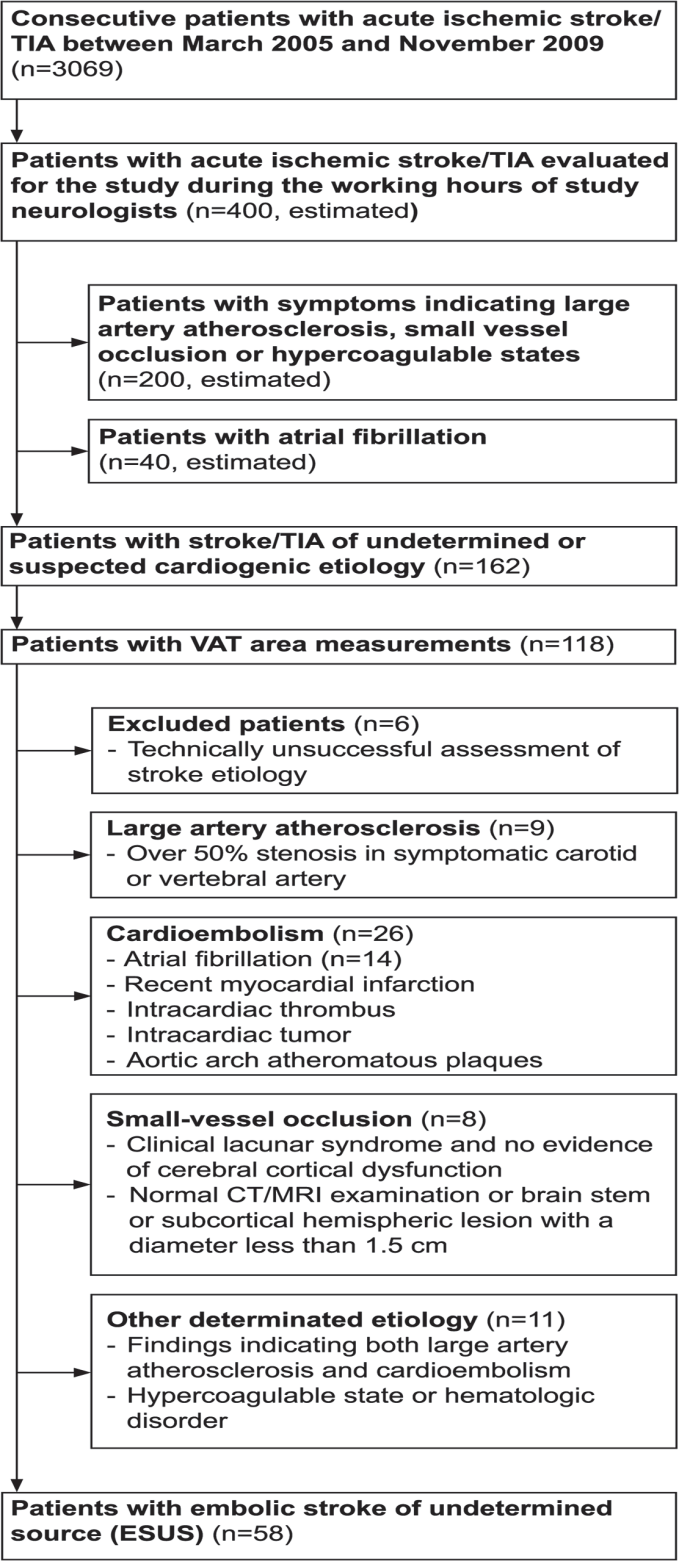
Flow chart of patient recruitment. Neurologists recruited consecutive patients with acute ischemic stroke/TIA with undetermined etiology or a suspicion of cardiogenic etiology. Stroke/TIA patients with atrial fibrillation were excluded. Patients who had undergone the assessment of visceral adipose tissue were included in the current study. Six patients were excluded from the study due to technically unsuccessful assessment of stroke etiology. The remaining patients were further categorized according to the modified TOAST classification, denoting five subtypes of ischemic stroke: 1) large-artery atherosclerosis, 2) cardioembolism, 3) small-vessel occlusion, 4) stroke of other determined etiology, and 5) embolic stroke of undetermined source (ESUS). The classification was updated by applying the more recent EAE recommendations for defining cardiac sources of embolism.

A flow chart describing the formation of the study population is presented in Fig. 1. Patients with ischemic stroke or transient ischemic attack (TIA) admitted to Kuopio University Hospital between March 2005 and October 2008 were evaluated as candidates for the study (EMBODETECT) [22, 23]. A total of 162 patients with stroke/TIA of undetermined or suspected cardioembolic etiology other than atrial fibrillation were recruited by the neurologists involved in the study as a part of their daily clinical work. The suspicion of cardiogenic etiology was based on the characteristic clinical symptoms and/or primary clinical signs, i.e., simultaneous or sequential strokes/TIAs in different arterial territories, hemorrhagic transformation, simultaneous emboli in other organs, decreased consciousness at stroke/TIA onset, isolated aphasia, or isolated visual-field defect. Patients with chronic or other previously known atrial fibrillation (AF) were excluded. Of 162 originally recruited patients the VAT measurements were performed on 118 patients. Furthermore, 60 patients were excluded due to a defined etiology for stroke (n = 54) or technically unsuccessful assessment of stroke etiology (n = 6). We modified the TOAST classification for cardioembolism, applying the more recent European Association of Echocardiography (EAE) recommendations for defining cardiac sources of embolism [24, 25]. Accordingly, 26 patients were found to have cardiogenic stroke. Large artery atherosclerosis was found in nine patients, small vessel occlusion in eight patients and other determined etiology in 11 patients. After profound etiologic investigations of stroke etiology, 58 patients fulfilled the criteria for ESUS and formed the final study population.

#### Reference population

We used three different reference populations. The first one was a large North American population published previously (n = 1160) [26], where the area of VAT was measured from a single CT slice at the level of the umbilicus. The ethnicity of the subjects in this reference population was 88% non-Hispanic white, 10% Hispanic and the remainder either Asian or African-American. The second reference population was a previously published local population from Kuopio University Hospital area consisting of healthy non-Hispanic white offspring of type 2 diabetic patients (n = 129) [27, 28]. In this study the area of VAT was measured from a single CT slice at the level of the fourth lumbar vertebra (L4). Both previously published reference studies used an attenuation range from −30 to −190 Hounsfield Units (HU) to measure adipose tissue. The third reference population is an unpublished local group of 18 healthy male subjects from the Kuopio University Hospital area. The VAT area was measured at the level of the L4 vertebra using an attenuation range of −30 to −190 HU. Increased VAT was defined as VAT above the mean+2SD in the North American reference population (>122.8 cm^2^ for females and >188.4 cm^2^ for men).

Detailed statistical comparison of the reference populations is presented in S1 Table. Noteworthy, men in the North American reference population and the local reference population 2 were coeval and had similar VAT area, whereas the local population 1 consisted of younger individuals with higher VAT when compared to the North American ones. Women in the North American reference population were older and had smaller VAT areas than in the local reference population 1 but there was no difference in BMI.

### Measurement of VAT

**Fig 2:**
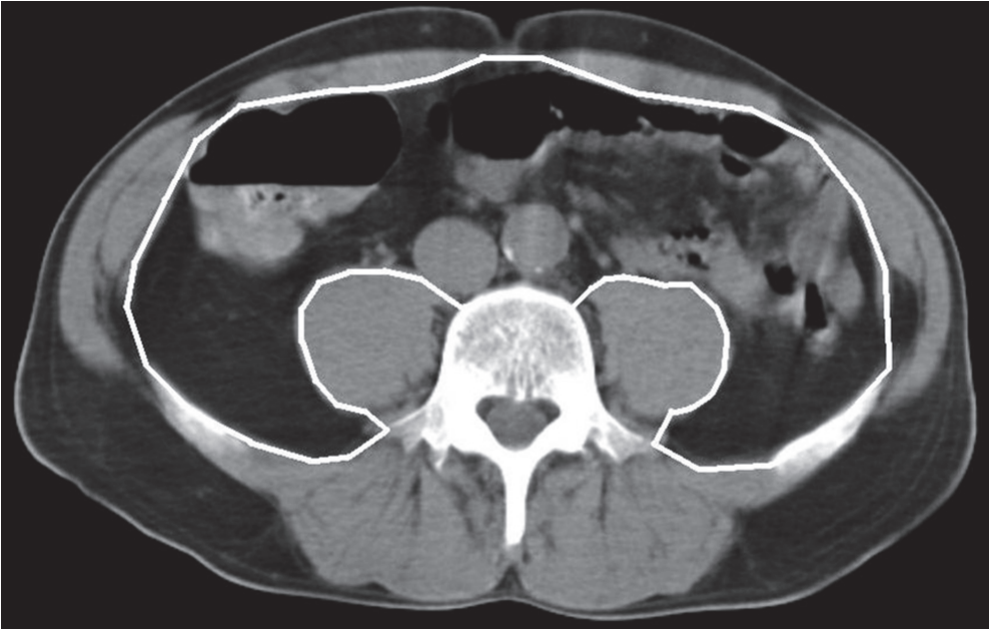
The image illustrates how the area of visceral adipose tissue (VAT) within the abdominal cavity (white line) was determined by CT scanning. Pixels with the density of adipose tissue between −30 and −190 Hounsfield Units (HU) were included in the VAT area calculated automatically by the CT software. In the illustrative case, the VAT area is 110 cm^2^.

VAT was measured with CT (Sensation 16; Siemens Medical Solutions, Forchheim, Germany). Scanning was performed at 120 kV, 190 mAs with a slice thickness of 10 mm. The radiation dose was calculated by the dose length product and it was 0.2 mSv, which equals around 8% of annual background radiation [29]. The image quality for the assessment of VAT was sufficient in all patients. The subjects were examined in the supine position with their arms stretched above their heads. The fourth lumbar vertebra was identified on the scout image and one axial slice from that level was obtained [30]. The VAT area was calculated by drawing a line within the muscle wall delineating the abdominal cavity and then computing the adipose tissue surfaces with an attenuation range from −30 to −190 HU [31]. All VAT area measurements were done by the same investigator (A.M.) guided by an experienced radiologist (J.M.). The measurement is illustrated in Fig. 2.

### Assessment of conventional risk factors

Subjects were dichotomized as never-smokers or as current/former smokers according to their self-reported smoking status. Subjects were dichotomized to have hypertension or diabetes if they were diagnosed either previously or during their hospital stay. BMI was determined by measuring the height and weight from lightly clothed patients without shoes. Based on BMI patients were categorized as normal weight (BMI <25 kg/m^2^), overweight (25 kg/m^2^ ≤ BMI <30 kg/m^2^) and obese (BMI ≥30 kg/m^2^). The fasting total cholesterol, high density lipoprotein (HDL)-cholesterol, low density lipoprotein (LDL)-cholesterol and triglyceride measurements were obtained the next morning after hospital admission. Body surface area (BSA) was calculated using the Mosteller’s formula [32].

### Statistical analysis

The associations between sex, smoking, hypertension, diabetes and VAT were studied using Independent Samples T-test. The effect size between aforementioned variables was studied using Cohen’s d (d) [33]. Pearson’s correlation coefficient was used to study correlations between VAT and age, total cholesterol, LDL-cholesterol, HDL-cholesterol, triglycerides, and BMI. Correlates that associated with VAT were included in the multiple regression analysis. Of the continuous variables, HDL-cholesterol, triglycerides and BMI were not normally distributed so they were log transformed for statistical analyses. Continuous variables are expressed as mean ± one standard deviation. In comparative analyses, age adjusted values were used. Categorical variables are presented as absolute values and percentages of the study population. p≤0.05 was considered statistically significant. SPSS 19.0 (1989−2010, SPSS Inc, Chicago, USA) and R version 2.14.0 (2011, The R Foundation for Statistical Computing) software were used for the statistical analyses.

## Results

The clinical characteristics of the stroke patients and the reference populations are summarized in Table 1. Over 75% of the patients with ESUS were men. Fifty-two percent and 21% of men, and 50% and 36% of women were overweight and obese, respectively. Eleven patients (19%) were on cholesterol-lowering medication. Women had higher mean HDL-cholesterol than men, 54 mg/dL (1.40 mmol/L) vs. 40 mg/dL, (1.04 mmol/L), d = 0.79, p = 0.037).

**Table 1:** Clinical characteristics of the study population and the reference populations.

	The study population		The North American reference population ^26^		The local reference population 1 ^27, 28^		The local reference population 2	
Subjects, n	58		1160		129		18	
Sex	75.9% men		55.2% men		45.7% men		100% men	
Ethnicity	100% non-Hispanic white		88% non-Hispanic white		100% non-Hispanic white		100% non-Hispanic white	
	**Men (n = 44)**	**Women (n = 14)**	**Men (n = 640)**	**Women (n = 520)**	**Men (n = 59)**	**Women (n = 70)**	**Men (n = 18)**	**Women (n = 0)**
Age, years	57.0±10.4	60.1±9.4	56.0±11.4	57.0±11.2	34.9±6.2 **	36.4±6.4 **	53.4±3.2 *	*NA*
VAT area, cm^2^	205.3±103.0	168.0±99.1	99.6±44.4 **	54.2±34.3 **	125.1±67.8 **	85.0±11.9 **	118.3±75.9 **	*NA*
BMI, kg/m^2^	27.5±4.2	29.8±4.9	27.8±3.9	25.6±4.8 **	26.0±3.5	26.3±5.4 *	25.0±3.1 *	*NA*
Total cholesterol, mg/dl	172.0±45.6	170.1±37.1	197.9±37.3 **	204.3±37.1 **	195.3±34.4 **	184.8±33.3	215.1±41.8 **	*NA*
HDL-cholesterol, mg/dl	40.4±11.6	54.2±21.8	44.3±12.8 *	62.3±16.0	44.1±8.5	53.0±10.8	59.9±17.6 **	*NA*
LDL-cholesterol, mg/dl	114.5±42.2	103.9±33.9	*NA*	*NA*	*NA*	*NA*	136.5±35.5 *	*NA*
Triglycerides, mg/dl	122.5±88.0	105.8±54.8	*NA*	*NA*	119.6±62.0	86.8±43.4	92.2±45.8	*NA*
Diabetes mellitus, n (%)	4 (9.1)	2 (14.3)	19 (3.0)	8 (1.5)	0 (0.0)	0 (0.0)	*NA*	*NA*
Hypertension, n (%)	24 (54.5)	8 (57.1)	225 (35.6)	146 (27.6)	*NA*	*NA*	*NA*	*NA*
Current or former smoker, n (%)	16 (26.3)	3 (21.4)	290 (45.3)	218 (41.9)	*NA*	*NA*	*NA*	*NA*

VAT, visceral adipose tissue; BMI, body mass index; HDL, high density lipoprotein; LDL, low density lipoprotein

* statistically significant difference at level p<0.05 compared to the same gender group in study population

** statistically significant difference at level p<0.01 compared to the same gender group in study population

**Fig 3:**
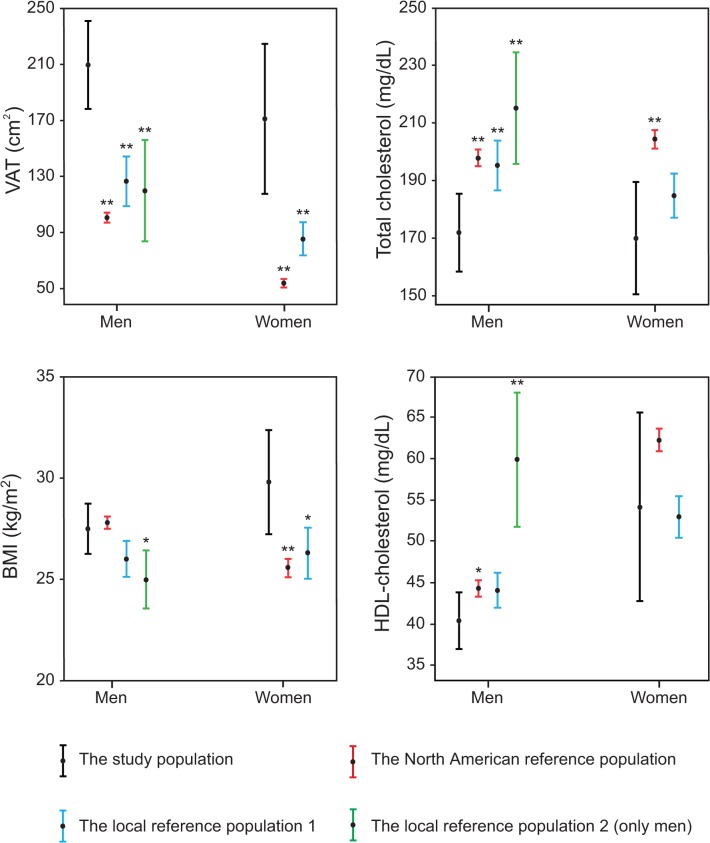
Age adjusted mean values and their 95% confidence intervals of visceral adipose tissue (VAT) and the conventional risk factors in patients with ESUS compared to the three control populations. Statistically significant differences between patients with ESUS and reference populations are marked by * (p<0.05) and ** (p<0.01).

Fig. 3 shows the differences in VAT, BMI, total cholesterol and HDL-cholesterol between patients with ESUS and the reference populations. In both male and female patients with ESUS the VAT area was almost or over twofold when compared to all the reference populations. In women, BMI was higher in the study population compared to both reference populations whereas in men there was a significant difference only when compared to the second local reference population. Unexpectedly, the mean total cholesterol of both men and women was lower in our study patients when compared to all three reference populations. Male stroke patients had significantly lower HDL-cholesterol compared to all reference populations whereas in the female group there was a significant difference only in comparison to the North American reference population.

**Fig 4:**
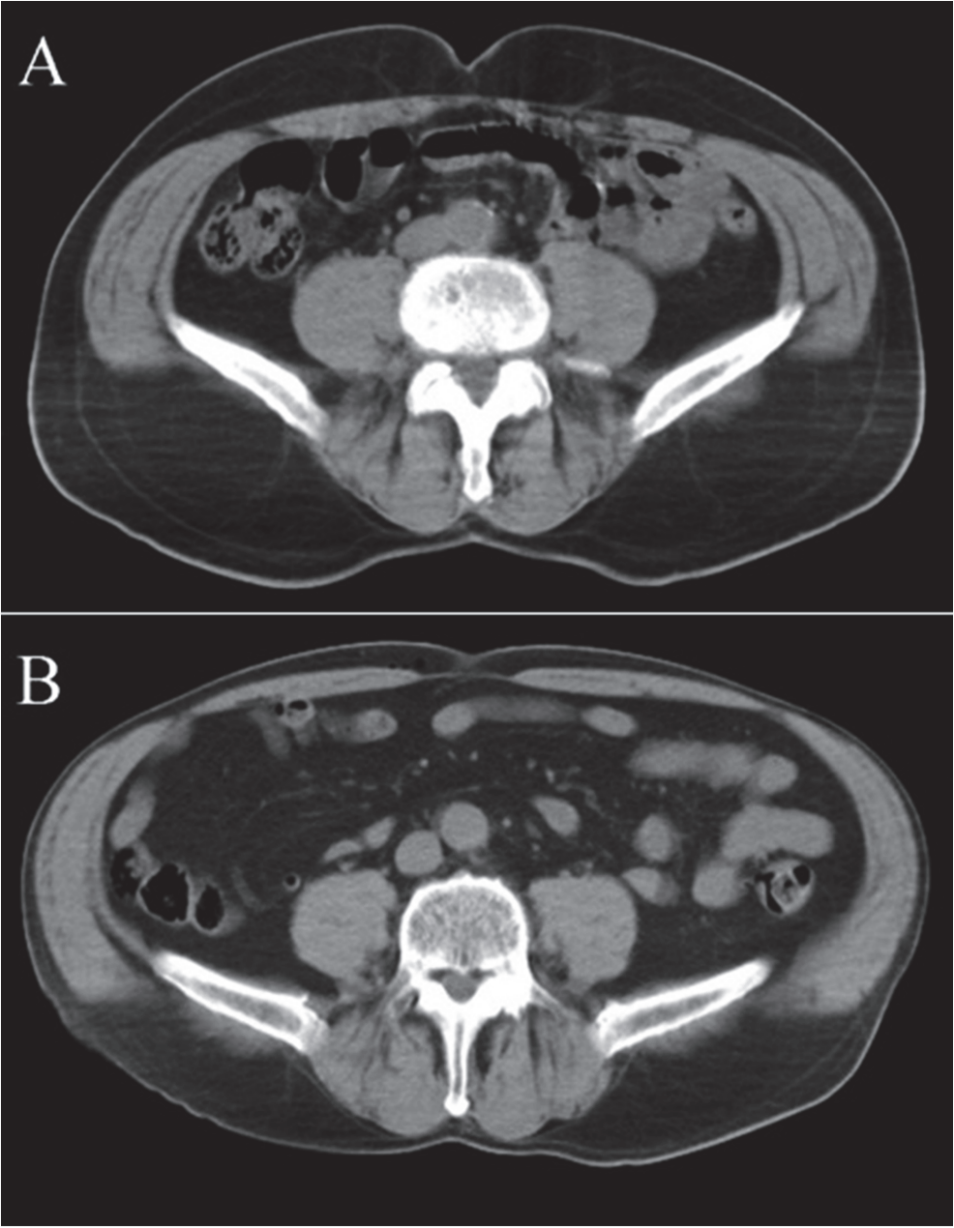
Patient examples with the same body mass index (BMI) and normal or increased (>188.4 cm^2^ for men) areas of visceral adipose tissue (VAT). (A) Male, age 69 years, BMI 25 kg/m^2^, VAT area 80 cm^2^. (B) Male, age 71 years, BMI 25 kg/m^2^, VAT area 230 cm^2^.

Half of the male patients and 57% of female patients had VAT area increased over the set upper limit of normality. Almost 80% of normal weight patients (BMI <25 kg/m^2^) and only 7% of obese (BMI ≥30 kg/m^2^) patients had normal VAT area. Though, 48% of male patients and 71% of female patients with overweight BMI (25 kg/m^2^ ≤ BMI <30 kg/m^2^) had normal VAT area. Patient examples of normal and increased VAT are presented in Fig. 4.

There were moderate positive correlations between VAT and BMI (r = 0.562, p<0.001) and VAT and BSA (r = 0.453, p<0.001). The number of patients with diabetes was small (n = 6) but this group of patients had larger VAT areas when compared to non-diabetics (319 cm^2^ vs. 182 cm^2^, d = 1.85, p = 0.001). In study patients, the VAT area was not associated with age or any of the cholesterols or triglycerides.

## Discussion

Our main finding was that over half of the patients with embolic stroke of undetermined source had increased VAT, and the mean VAT in the whole group was two-fold larger when compared to three different reference populations, one Northern American and two local ones. Moreover, increased VAT was more commonly found than any single conventional risk factor for stroke. Interestingly, in male patients, VAT was significantly higher compared to all of the reference populations although BMI didn’t show similar trend. Patients were determined to have acute ESUS after exclusion of CAA, AF or other high risk sources for cardiogenic embolism. To the best of our knowledge, the area of VAT has not been previously studied in this patient group.

Our results indicate that the increased amount of visceral type fat might be a risk factor for ESUS. The pathophysiology of ESUS is largely unknown and the role of conventional risk factors is less clear than in stroke of a defined etiology [34]. Similarly, many of our patients were lacking all conventional risk factors and only moderate positive correlations were found between VAT and diabetes, BMI, decreased HDL cholesterol, and BSA. There was no difference in the BMI between men in our study populations and two reference populations even if a highly significant difference in VAT was found. Moreover, more than a half of the overweight patients had a normal VAT area. Therefore, VAT measurements cannot be replaced by BMI. BMI is not sensitive to the differentiation of body fat/muscle ratio [35] or the variable distribution of visceral and subcutaneous fat.

The importance of body fat distribution has been recognized for more than 60 years [36]. Individuals with upper abdominal, central or android obesity are at a greater risk of cardiovascular diseases than those with gluteofemoral or peripheral obesity. It has been shown that the response to insulin and other hormones differs between visceral and subcutaneous adipose tissue, and that VAT is metabolically more active than subcutaneous adipose tissue. It secretes several pro- and anti-inflammatory factors that are associated with endothelial damage [37], modulates the inflammatory state and procoagulant response via increased secretion of procoagulant mediators such as plasminogen activator inhibitor-1 (PAI-1), an endogenous inhibitor of fibrinolysis [38–41], tissue factor (TF) [40–42], and via enhanced platelet activity [43]. The potential role of enhanced procoagulative tendency is further strengthened by the fact that increased epicardial fat, which has been shown to have a strong correlation with the amount of visceral adipose tissue, has been reported to be strongly associated with idiopathic venous thrombosis (VTE), even after adjustment for the established risk factors for atherosclerosis [44]. VTE and intracardiac thrombosis are known to share similar constituents (predominantly fibrin and erythrocytes with fewer platelets) and pathogenic factors (stasis, altered vessel wall and increased activity of coagulation).

VAT also predisposes patients to atherosclerosis [45, 46] and it has been shown to be independently associated with paroxysmal and persistent atrial fibrillation [47]. In our study, patients with CAA, previously known AF or AF diagnosed by telemetry or 24-h Holter recording during or immediately after hospital stay were excluded, but the presence of silent atrial fibrillation cannot be totally ruled out. In conclusion, the increased visceral adipose tissue could expose a subject to the risk of ESUS both by 1) promoting an inflammatory and prothrombotic state and 2) predisposing to latent atrial fibrillation.

We used a single CT slice for VAT area assessments because it has been shown to be an accurate quantitative estimate of VAT [48]. CT is the most reproducible method for the assessment of the adipose tissue area along with MRI [48, 49]. The reproducibility of CT-assessed VAT has been shown to be excellent with an error of reproducibility of less than 1% [49–51]. Ultrasound imaging is a radiation-free method but it yields inconsistent results [52]. MRI also enables radiation-free assessment of the VAT area, however, with poorer availability and higher costs. The clinical value of VAT in the risk stratification of stroke remains to be evaluated in future. Determination of the VAT might improve risk stratification and the allocation of secondary prevention resources, the benefit of which could exceed the harm caused by the small amount of radiation (0.2 mSv) caused by VAT CT imaging.

There are limitations of the present study. The most important limitation is the reference population. Only two small local reference populations (n = 129 and n = 18) with similar VAT quantification methods were available due to radiation-related restrictions. These two control populations consisted of healthy individuals from the Kuopio University Hospital area. The latter consisted only of males, who were considerably younger than our patients. Additionally, a large population from North America (n = 1160) was used as a reference. In this North American population the distribution of age and sex was similar, but the ethnicity was somewhat different (88% non-Hispanic white, 10% Hispanic and the remaining two percent either Asian or African-American vs. 100% non-Hispanic white in our homogenous patient group). However, in previous studies no difference in the VAT area at the level of L4/L5 vertebra has been found between Hispanic and non-Hispanic white populations [53] and the vast majority of non-Hispanic whites in Northern America trace their origins to the Northwestern Europe [54]. Most importantly, the main results were similar regardless of which of these three populations was used. Secondly, in the North American reference population, the area of VAT was measured from a single CT slice at the level of the umbilicus, whereas in our study and in the local reference studies a skeletal landmark was used. Practically, these two landmarks are at the same position as shown in a previous study [55]. However, regarding to the North American reference population we had access only to the published data so a profound methodological comparison of VAT measurement could not be performed. Thirdly, 19% of our patients were on cholesterol-lowering medication, which could interfere the interpretation of the role of blood lipids as risk factor. However, the share of patients using cholesterol-lowering medication in the North American reference population was similar [56]. Information about the use of cholesterol-lowering medication in the local reference populations was not available. The fourth limitation concerns the relatively small number of patients with ESUS and especially the number of female patients. This is due to the strict criteria of including only patients with ESUS after excluding CAA or a cardiogenic source for stroke.

In conclusion, patients with ESUS have highly increased VAT compared to unmatched reference population. Increased VAT may serve as a pathogenic organ for promoting the formation of thrombus and may thus contribute to the etiology of the current stroke and increase the risk of future strokes in this selected stroke population. Recognizing individuals with an increased amount of VAT could theoretically help in preventing stroke recurrence if appropriate treatment is launched. Further clinical studies are needed to clarify whether a reduction in VAT could also decrease the risk of recurrent stroke.

## Supporting Information

S1 Table Statistical comparison of the reference populations. (PDF)
