# Supplementary material for: Increased Visceral Adipose Tissue as a Potential Risk Factor in Patients with Embolic Stroke of Undetermined Source (ESUS)
Source: PLoS One. 2015 Mar 10;10(3):e0120598. doi: 10.1371/journal.pone.0120598 (PMC4354901; doi:10.1371/journal.pone.0120598)
Supplement: S1 Table — (PDF) [file pone.0120598.s001.pdf]

Table S1. Statistical comparison of the reference populations.

	**The North American reference population** [26]		**The local reference population 1** [27, 28]		**The local reference population 2**	
Subjects, n	1160		129		18	
Sex	55.2% men		45.7% men		100% men	
Ethnicity	88% non-Hispanic white		100% non-Hispanic white		100% non-Hispanic white	
	**Men (n=640) A**	**Women (n=520) B**	**Men (n=59) C**	**Women (n=70) D**	**Men (n=18) E**	**Women (n=0)**
Age, years	56.0±11.4 *A vs. C* ** *A vs. E ns*	57.0±11.2 *B vs. D* **	34.9±6.2 *C vs. A* ** *C vs. E* **	36.4±6.4 *D vs. B* **	53.4±3.2 *E vs. A ns* *E vs. C* **	*NA*
VAT area, cm^2	99.6±44.4 *A vs. C* ** *A vs. E ns*	54.2±34.3 *B vs. D* **	125.1±67.8 *C vs. A* ** *C vs. E ns*	85.0±11.9 *D vs. B* **	118.3±75.9 *E vs. A ns* *E vs. C ns*	*NA*
BMI, kg/m^2	27.8±3.9 *A vs. C* ** *A vs. E* *	25.6±4.8 *B vs. D ns*	26.0±3.5 *C vs. A* ** *C vs. E ns*	26.3±5.4 *D vs. B ns*	25.0±3.1 *E vs. A* * *E vs. C ns*	*NA*
Total cholesterol, mg/dl	197.9±37.3 *A vs. C ns* *A vs. E ns*	204.3±37.1 *B vs. D* **	195.3±34.4 *C vs. A ns* *C vs. E* *	184.8±33.3 *D vs. B* **	215.1±41.8 *E vs. A ns* *E vs. C* *	*NA*
HDL-cholesterol, mg/dl	44.3±12.8 *A vs. C ns* *A vs. E* **	62.3±16.0 *B vs. D* **	44.1±8.5 *C vs. A ns* *C vs. E* **	53.0±10.8 *D vs. B* **	59.9±17.6 *E vs. A* ** *E vs. C* **	*NA*
LDL-cholesterol, mg/dl	*NA*	*NA*	*NA*	*NA*	136.5±35.5	*NA*
Triglycerides, mg/dl	*NA*	*NA*	119.6±62.0 *C vs. E ns*	86.8±43.4	92.2±45.8 *E vs. C ns*	*NA*

VAT, visceral adipose tissue; BMI, body mass index; HDL, high density lipoprotein; LDL, low density lipoprotein

* Statistically significant difference at level $p<0.05$, ** statistically significant difference at level $p<0.01$
